# State of Diseases in the Bristol Dispensary

**Published:** 1801-04

**Authors:** W. D. Rolfe

**Affiliations:** Apothecary and Man-midwife to the Bristol Dispensary


### *To the Editors of the Medical and Phyſical Journal.*

GENTLEMEN,

BEING a conſtant reader of your valuable Journal, and a well wiſher to every attempt at improvement in Medical Science, I obſerved with pleaſure the account given in one of your late numbers, of "Diſeaſes in the Liverpool Diſpenſary." As you ſolicit ſimilar communications, I am induced to ſend you the following Account of Caſes that have occurred at the Briſtol Diſpenſary; if you think it worth inſertion, it is much at your ſervice.

*Cases occuring at the Bristol Dispensary, from January* 1, *to December* 31, 1800.

| | | | |
|---|---|---|---|
| Aſthma | 18 | Conſumption | 64 |
| Typhus Fever | 176 | Scrophula and Scorbutic | 16 |
| Dyſenteria and Diarrhœa | 114 | Fluor Albus | 5 |
| Puerperal Fever | 23 | Sarcocele | 1 |
| Menſtrual Complaints | 6 | Peripneumonia | 42 |
| Small-pox | 12 | Opthalmia | 6 |

Continued

| | | | |
|---|---|---|---|
| Continued Fever - - - | 183 | Bilious Complaints - - | 17 |
| Inflammation and Contufion | 8 | Stone and Gravel - - - | 6 |
| Dropfy - - - - - - - | 33 | Afthenia - - - - - - | 3 |
| Hæmoptæ - - - - - - | 9 | Cough - - - - - - | 4 |
| Podagra - - - - - - - | 5 | Meafles - - - - - - - | 10 |
| Eryfipelas - - - - - - | 8 | Prolapfus Uteri - - - | 1 |
| Uterine Hæmorrhage - - | 14 | Retrovertio Uteri - - - | 1 |
| Stomach Complaints - - | 53 | Laceration of the Perineum | 1 |
| Paralyfis - - - - - - - | 10 | Fright - - - - - - - | 2 |
| Pleuretic - - - - - - | 18 | Jaundice - - - - - - | 1 |
| Hæmorrhoidals - - - | 2 | Suppreffion of Urine - - | 2 |
| Convulfions - - - - - - | 3 | Epilepfy - - - - - - - | 2 |
| Impotence - - - - - | 1 | Painter's Colic - - - - | 1 |
| Complaints of the Head - | 3 | Intermittent Fever - - | 3 |
| Hectic Fever - - - - - | 11 | Putrid Fever - - - - - | 8 |
| Nervous Complaints - - | 10 | Quinfy - - - - - - - | 3 |
| Acute Rheumatifm - - - | 10 | Spafmodic Affections - - | 4 |
| Diabetes - - - - - - | 3 | Cholera Morbus - - - | 1 |
| Worms - - - - - - - | 2 | Difeafed Kidney - - - | 1 |
| Abortion - - - - - - | 8 | Rickets - - - - - - - | 2 |
| Chronic Rheumatifm - - | 9 | Milk Fever - - - - - - | 2 |

It may be neceffary to obferve, that the *Bristol Dispensary* was originally eftablifhed for the benevolent purpofe of attending fuch of the poor of the city, *at their own houses*, as from various circumftances are precluded from becoming *in* or *out* patients of the Briftol Infirmary, (to which therefore the charity is juftly confidered as an auxiliary,) but from this caufe the numbers who receive benefit from it are confiderably lefs than at other charitable inftitutions under the fame *name*. I am, &c.

W. D. ROLFE,

Apothecary and Man-midwife to the Briftol Difpenfary.

*March* 15, 1801.

*N. B.* I intended fending the ftate of this year's patients monthly, and alfo a ftatement of fome cafes of Dropfy, &c. treated with Digitalis, which I have been prevented doing by ficknefs of myfelf and family, but will now give two months ftate of the patients; and endeavour, for the future, to fend it monthly, with the ftate of cafes above mentioned.

*State of the Patients in the Briftol Difpenfary, from the 1ft of January to the 1ft of March,* 1801.

| | | | |
|---|---|---|---|
| Typhus Fever - - - - | 87 | Uterine Hæmorrhage - - | 4 |
| Dropfy - - - - - - | 14 | Rheumatifm - - - - - | 10 |
| Mania - - - - - - - | 1 | Spafmodic Affections - | 3 |
| Phthifis Pulmonalis - - | 3 | Scrophula and Scorbutic - | 7 |
| Bowel Complaint - - - | 16 | Simple Fever - - - - - | 16 |
| Confumption - - - - - | 10 | Peripneumonia - - - | 28 |
| Cough and Dyfpnœa - - | 9 | Afthenia - - - - - - - | 3 |

Leucorrhœa

